# Hepatocellular Carcinoma in Liver Cirrhosis: Surgical Resection versus Transarterial Chemoembolization—A Meta-Analysis

**DOI:** 10.1155/2015/696120

**Published:** 2015-01-06

**Authors:** Teodor Kapitanov, Ulf P. Neumann, Maximilian Schmeding

**Affiliations:** Department of General, Visceral and Transplantation Surgery, University Hospital Aachen, RWTH Aachen, Pauwelstraße 30, 52074 Aachen, Germany

## Abstract

We compare the value of TACE to liver resection for patients with BCLC stage A and B HCC. For patients with HCC in cirrhosis LT is the treatment of choice. TACE represents the current standard for unresectable BCLC stage B patients not eligible for LT. Recently liver resection for HCC and significant cirrhosis has become increasingly popular. A systematic search of the literature and meta-analysis was conducted to identify studies, reporting short- and long-term results of hepatic resection versus TACE for HCC treatment. The data were analyzed regarding the odds for 30-day mortality and hazard ratio for overall-survival. 12 studies comparing short- and long-term outcome of HR versus TACE for HCC were identified. Peri-interventional mortality and overall survival were investigated. Peri-interventional mortality was higher for surgical resection (n.s.), and overall-survival was significantly better for surgically treated patients at one year (*P* = 0.002) and 3 years (*P* ≤ 0.00001). The hazard ratio of overall-survival for all twelve studies was 0.70 (*P* = 0.0001) and significantly in favor of surgical treatment. Although large RCTs are missing and the available data are limited and not homogeneous a reappraisal of the current treatment guidelines should be considered based on the superior long-term outcome for surgically treated patients.

## 1. Introduction

Hepatocellular carcinoma (HCC) most often develops in patients with liver cirrhosis. Liver transplantation (LT) is the treatment of choice for those patients with limited tumor load within the Milan criteria and younger than approximately 70 years [1, 2]. Despite increasing numbers of HCC patients on the waiting lists for LT since the introduction of the MELD score based allocation system the universal shortage of adequate liver grafts complicates treatment options. Based on the Barcelona Clinic Liver Cancer (BCLC) criteria surgical resection should be attempted for patients with HCC if portal hypertension is absent and serum-bilirubin values are normal and patients are not eligible for LT [3]. Transarterial chemoembolization of HCC is currently regarded as the standard treatment for stage B patients.

For tumors of diameter ≤ 3 cm radiofrequency ablation is recommended in current guidelines (EASL, AASLD, and JSH) [4]. Surgical resection of HCC in cirrhotic liver tissue has been regarded as a risky procedure incorporating both elevated perioperative morbidity and mortality and limited long-term benefit [5]. Therefore surgical treatment has been restricted to patients with rather limited disease, meaning that only patients with relatively small tumors, no portal hypertension, Child-Pugh A status, and normal serum bilirubin values have been candidates for hepatic resection.

While both surgical techniques and experience and anesthesiological and intensive care management have improved significantly during the last 15 +/− years, the limits of indication for liver resection have been expanded. Several specialized hepatobiliary surgeons have demonstrated encouraging results for surgical resection of BCLC stage A and B tumors [6, 7]. Up to this date a number of studies have shown favorable long-term results for surgical treatment compared to TACE [8, 9].

However, most of these investigations are of nonrandomized, single-center character incorporating rather a heterogeneous patient collective.

Surgical resection and radiofrequency ablation for HCC patients have been compared in rather a large randomized trial of 230 patients within the Milan criteria (BCLC stage A) indicating a favorable outcome for surgically treated patients [10]. A recent meta-analysis incorporates three randomized and 25 nonrandomized trials investigating this issue; the results confirm the long-term superiority of surgical treatment [11].

As RFA can only be recommended for tumors ≤ 3 cm in diameter TACE is currently advocated for larger or multiple lesions (BCLC stage B).

A comparably profound data analysis does not exist for the important question whether to perform TACE or surgery on BCLC stage A and B HCC patients. This meta-analysis aims to analyze the available studies in order to clarify the picture and shed some more light upon the important question whether or not to expand the limits of surgical resection in cirrhotic patients with HCC.

## 2. Methods

**Figure 1:**
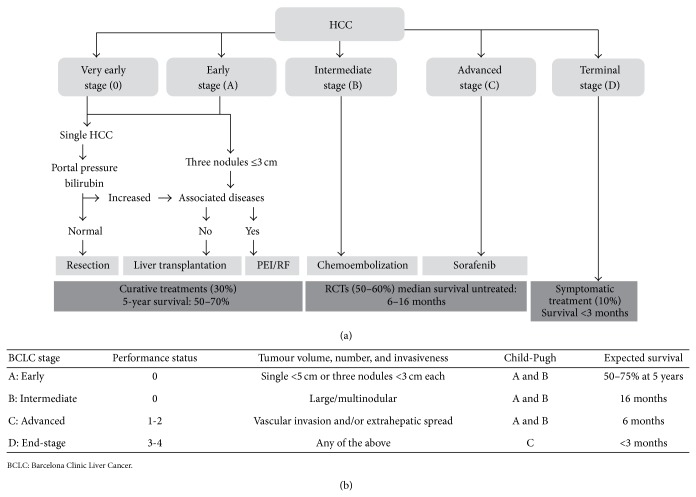
((a), (b)) Strategy for staging and treatment assignment in patients diagnosed with HCC according to the BCLC proposal [12].

Meta-analysis according to the Cochrane Collaboration guidelines concerning short- and long-term outcome of TACE versus surgical resection for HCC was performed. Different follow-up intervals, that is, 30-day or in-hospital mortality, one, three, and five years after the respective procedures were investigated. Strategy for staging and treatment assignment in patients diagnosed with HCC according to the BCLC proposal [12] is shown in Figure 1.

### 2.1. Literature Search

Employing six electronic bibliographical databases (PubMed, Medline, Embase, CINAHL, BIOSIS, and Cochrane Database of Systemic Reviews) the literature was screened for studies investigating concurrently hepatic resection and TACE. For the search the following keywords were used: hepatic resection, transcatheter arterial chemoembolization, hepatocellular carcinoma, overall-survival, and short- and long-term overall-survival. A manual search was performed using the references in reviews and articles. The selection was limited to articles published in English.

### 2.2. Study Selection Criteria

Inclusion criteria were defined as follows: published comparative studies reporting short- and long-term overall-survival outcomes. Studies reporting less than 30 patients were omitted from the analysis. Data extraction and comparison were carried out and checked for accuracy by two independent reviewers. Disagreements were resolved by consensus. Studies not containing extractable comparative data were not included. Publications presenting outcome by Kaplan-Meier survival curves in which the exact number of annual survivals could not be determined were not considered.

**Figure 2:**
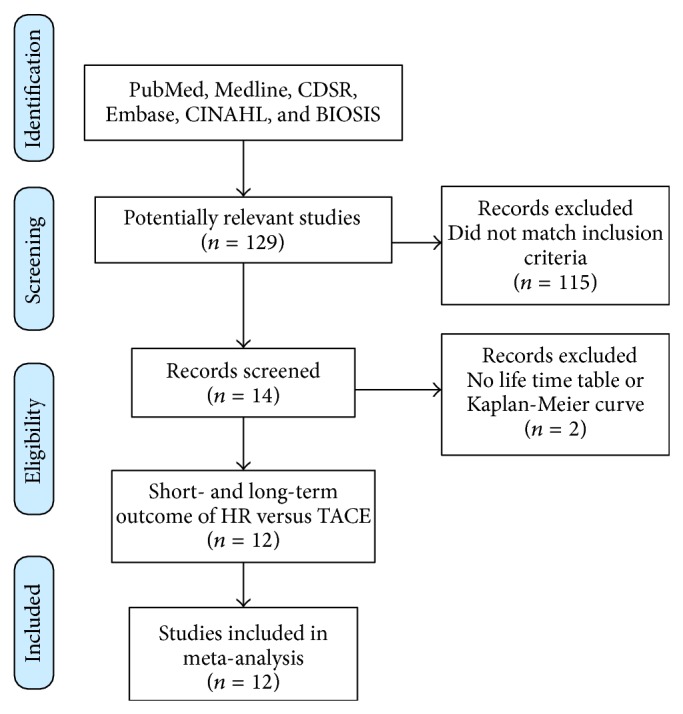
PRISMA flowchart diagram of search strategy.

The literature search identified a total of 129 publications. Fourteen articles were considered potentially relevant. Of these, 12 full-text papers reporting on short- and long-term outcome data after concurrent HCC treatment by either hepatic resection or TACE met the criteria for inclusion (see flowchart in Figure 2). Most studies were retrospective and single-center and all were not randomized. Patient collectives were heterogeneous and very variable in size. The characteristics of these publications are listed in Table 1.

### 2.3. Data Extraction and Analysis

All studies identified in our literature search reported the short- and long-term survival in the form of Kaplan-Meier curves. The number of patients event-free at each time point within a Kaplan-Meier curve is known and can be used to estimate the amount of censoring in a trial [24]. The methods to extract and calculate these statistics data have been described in detail by Tierney et al. [25] and Parmar et al. [26]. A calculation spreadsheet in Microsoft Excel was developed to obtain the observed minus expected events (O-E), the variance V, the hazard ratio HR, the log hazard ratio, and its standard error SE for each individual trial. Statistical analysis was undertaken using Review Manager software version 4.2.7 (the Cochrane Collaboration, Oxford, UK). The end points of this meta-analysis were 30-day or in-hospital mortality and short- and long-term overall-survival. The effect measures for 30-day mortality were described in odds ratios (ORs) and the overall-survival rates were expressed as hazard ratios (HRs). Random effects model was used because of heterogeneity among the studies. Meta-analysis was displayed graphically as “forest plots.” Heterogeneity was explored using chi-squared test. *I* ^2^ value was calculated to measure and quantify heterogeneity. Funnel plot (Figure 5) was used to examine reporting bias and heterogeneity in the results of meta-analyses. Statistical significance of the overall result was expressed with the probability value (*P* value). The result was regarded as statistically significant if *P* < 0.05.

## 3. Results 

### 3.1. 30-Day Mortality

**Figure 3:**
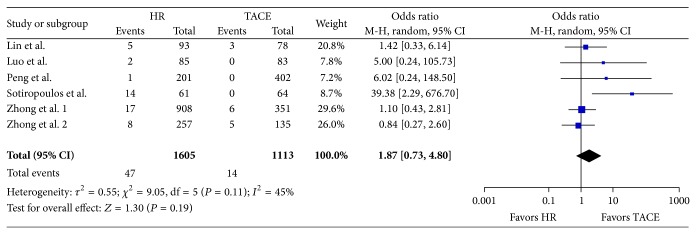
30-day mortality.

Six studies reported on 2,718 patients (HR *n* = 1.605, TACE *n* = 1.113). Odds ratios with 95% confidence intervals of the individual studies and in meta-analytic random effects model are shown in a forest plot in Figure 2. The 30-day mortality was higher in the hepatic resection group (OR, 1.87; 95% CI, 0.73 to 4.80; *P* = 0.19) but was not significantly in favor of TACE. The chi-squared test *I* ^2^ = 45% showed moderate heterogeneity (Figure 3).

### 3.2. Overall-Survival

The overall-survival was based on twelve trials incorporating a total of 9.116 patients (HR *n* = 5.394, TACE *n* = 3.722). The hazard ratios for short- and long-term overall-survival rates across the twelve trials were in favor of hepatic resection at one year (HR, 0.62; 95% CI, 0.46 to 0.85; *P* = 0.002) and at three years (HR, 0.59; 95% CI, 0.51 to 0.69; *P* ≤ 0.00001). At five years data incorporating 3.675 patients (HR, 1.07; 95% CI, 0.71 to 1.61; *P* = 0.75) showed no significant difference. The pooled estimates for hazard ratio of overall-survival for all studies among the entire follow-up period were HR, 0.70, 95% CI, 0.60 to 0.83, *P* = 0.0001, and were significantly in favor of the surgical procedure. The heterogeneity test showed high heterogeneity.

## 4. Discussion

The treatment of hepatocellular carcinoma is often complex with various medical disciplines involved. As the majority of patients with HCC suffer from liver cirrhosis surgical resection is limited due to the risk of inducing postoperative liver failure. Liver transplantation (LT) has clearly been demonstrated to offer the best chances of long-term survival [1]. This treatment, however, cannot be offered to all patients due to organ shortage. Age limitations and tumor dimension criteria (Milan/San Francisco/up-to-seven/and others) have been developed in order to allocate the scarce resources to the most adequate recipients [1, 27, 28]. For many patients who are not eligible for LT local ablation represents the current standard of care. Radiofrequency-induced tumor ablation (RFA) is advocated for cirrhotic patients with tumors no larger than 30 mm in diameter while transarterial chemoembolization (TACE) should be performed for larger or more diffuse tumors. While RFA has been evaluated quite extensively in large meta-analyses the value of TACE has not been clearly defined when compared to hepatic resection [29–31]. With increasing experience and improved surgical strategies, the limits of liver surgery in cirrhotic patients have been expanded in recent years leading to extended resection indications. Specialized HPB centers have demonstrated encouraging results for BCLC stage A and B patients [7]. Based on these recent findings the question has been raised if the current EASL standards which advocate RFA BCLC stage A and TACE for stage B HCC patients, respectively, need to be revised [4]. Therefore the intention of this meta-analysis was to compare the short- and long-term results of hepatic resection versus TACE in BCLC stage A and B patients.

The results of our literature research underline the heterogeneity of the available data. While 12 studies could be integrated into our survival analysis, only six studies demonstrated peri-interventional morbidity information. Further in-depth evaluation of potential prognostic parameters such as tumor size was complicated by the variety of classifications applied in the different studies.

Although the available literature is limited and prospective studies are rare our investigation draws a rather clear-cut picture. Based on the currently published information liver resection shows significantly improved long-term survival compared to TACE in cirrhotic patients with BCLC stage A and B HCC. The pooled estimate hazard ratio of the overall-survival was in favor of hepatic resection, 0.70 (95% CI, 0.60 to 0.83, *P* = 0.0001). Peri-interventional mortality had an odds ratio of 1.87 (95% CI, 0.73 to 4.80; *P* = 0.19). As expected 30-day peri-interventional mortality is significantly higher for surgically treated patients than for TACE patients. However, despite this short-term effect long-term survival is significantly improved for surgically resected patients. Despite this straightforward message several limitations have to be taken into account. All liver resections incorporated into this meta-analysis have been performed in highly specialized HPB surgical units. Most of the data are of retrospective and of nonrandomized nature, generating a potential bias that has to be respected when interpreting the results. The study by Luo et al. [18], a radiologic prospective nonrandomized investigation, demonstrates that TACE may serve as a potential selection tool for HCC patients who profit most from liver resection. In this study, patients who displayed good tumor response to TACE showed improved oncological outcome after liver resection following the TACE.

According to recent experiences of various groups it may be suggested that TACE serves to discriminate the patients with favorable tumor biology from the ones for whom all types of available treatment options offer merely dismal prognosis [18, 32, 33].

This theory may be supported by the fact that despite significantly improved 1- and 3-year survival figures 5-year survival was not statistically different for patients treated with TACE versus resection. For one reason, patient numbers were considerably smaller in the 5-year survival analysis than in the 1- and 3-year data pool. On the other hand, it may be suggested that those patients who were successfully treated by TACE for longer than three years were treated sustainably with very low risk of tumor recurrence.

Recently, a large prospective multicentre trial demonstrated clear superiority for hepatic resection when compared to TACE and RFA for patients with Child-Pugh stage A and B liver cirrhosis and stage II HCC (JIS scores 1 and 2) [22].

In 2012 Peng et al. [19] demonstrated that even for patients with portal venous tumor thrombus liver resection improves long-term survival compared to TACE as long as tumor thrombosis was confined to the liver. This effect vanished in the presence of extensive tumor thrombosis into the portal venous confluence and the superior mesenteric vein.

**Figure 4:**
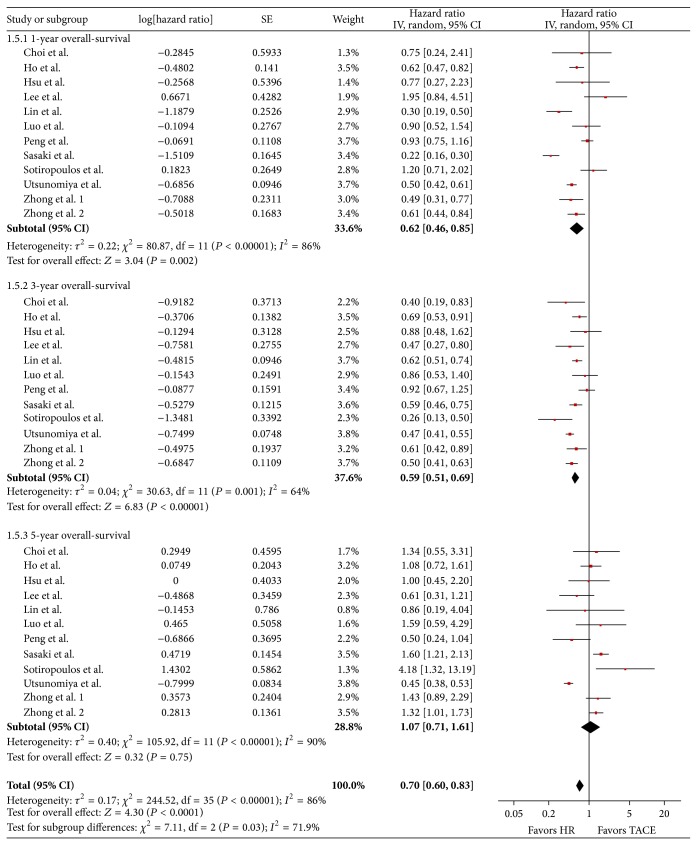
Forest plot illustrating subgroup analysis of short- and long-term overall-survival comparing hepatic resection to TACE. The center of each square represents the hazard ratio for individual trial and each horizontal line represents its 95% CI. The size of the box is directly related to the “weighting” of the study. The center of the diamond represents the pooled hazard ratio and the width represents its 95% CI. For each subgroup (1, 3, and 5 years), the sum of the statistics is represented by the first three diamonds. The last diamond illustrates the overall result of the meta-analysis.

**Figure 5:**
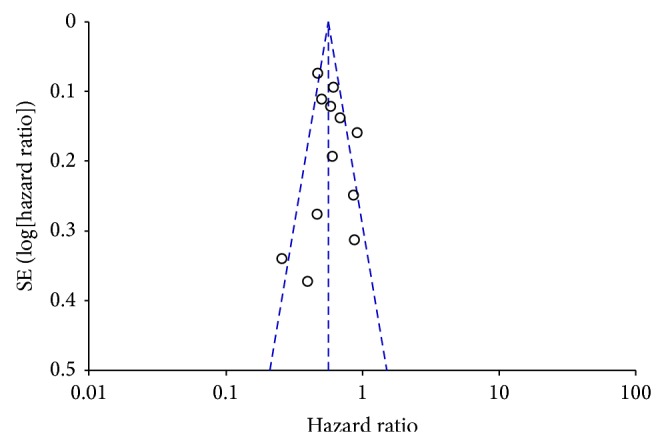
Funnel plot at 3-year overall-survival depicting the distribution of hazard ratios for the 12 studies included in the meta-analysis. The outer dashed lines indicate the triangular region within which 95% of studies are expected to lie in the absence of reporting biases and heterogeneity. The solid vertical lines correspond to no intervention effect.

The largest published analysis on this topic stems from a Chinese group and was published in 2014 by Zhong et al. [8]. The authors demonstrate clear superiority for hepatic resection versus TACE in terms of patient survival. Despite a rather heterogeneous patient collective the total number of 1259 that included individuals from a single regional database is impressive. The vast majority of cases are hepatitis-B positive and therefore are not typical for western HCC patient collectives. The study is somewhat limited by the fact that mean patient age and tumor size were both greater in the TACE group, a fact that is certainly attributable to preinterventional patient selection, a major drawback of retrospective investigations. For this reason matched-pair analysis was performed between TACE and resection patients with identical demographics confirming the positive overall results for surgically treated patients. AFP values ≥ 400 ng/mL, macrovascular invasion, and portal hypertension were identified as significantly negative prognostic parameters in multivariate analysis for both treatment modalities. However, even for these “high recurrence risk” patients hepatic resection offered significantly better survival than TACE (Figure 4).

As mentioned above there are some limitations of this meta-analysis. A patient selection bias in selecting the choice of treatment cannot be ruled out in this meta-analysis. In-depth investigation of the available data is complicated by the fact that different parameters and classifications are employed by various authors. It is therefore hard to generate substantial connections between outcome and potentially relevant parameters such as tumor size/number of tumor nodules/Child-Pugh stage. We have tried to elucidate the picture by analyzing outcome with respect to tumor size and liver function (see Table 1); however, small numbers clearly limit our results. For example, the study from Choi et al. incorporated similar numbers of tumors < 3 cm for both TACE and resection treatment; for tumors > 3 cm, however, TACE was employed almost three times more frequently [13]. Tumor size, on the contrary, was often greater for surgically treated patients in the large study by Zhong et al. [8].

Based on the currently published data a more aggressive surgical approach in the treatment of both BCLC stages A and B HCC seems justified. However, stratified prospective studies on this important and controversial issue are needed in order to consolidate the findings of this meta-analysis.

## Figures and Tables

**Table 1:** Demographics and specific results of all studies included in this meta-analysis.

Reference	Year	Inclusion period	Country	Number of patients (HR/TACE)	M/F (HR/TACE)	Mean age (years)	Mean AFP (ng/mL)	Child-Pugh A B C	Tumor size (cm)	Bilirubin total (mg/dL)	Albumin (g/dL)	Tumor number (HR/TACE)
Choi et al. [13]	2013	2003–2008	South Korea	36/107	34/2 86/21	54.3 ± 8.6 61.2 ± 9.3	—	—	≤3 19/17 ≥3 29/78	—	4.05 ± 0.5 3.93 ± 0.48	2 : 30/2 : 90 3 : 6/3 : 17
Ho et al. [14]	2009	1981–2000	Taiwan	294/367	240/54 290/77	57.0 ± 11.8 60.1 ± 12.3	8216 ± 55270 13464 ± 65879	229/234 13/49 0/9	5.0 ± 3.5/5.3 ± 3.5	1.2 ± 1.9 1.3 ± 1.4	3.8 ± 0.5 3.5 ± 0.5	—
Hsu et al. [15]	2012	2001–2007	Taiwan	112/73	76/39 —	60.3 ± 11.7 61.9 ± 11.7	≥200 30/13 ≤200 82/60	—	2.48 ± 0.69 2.34 ± 0.74	0.81 ± 0.33 0.91 ± 0.45	3.98 ± 0.45 3.87 ± 0.41	S: 41/S: 83 M: 32/M: 29
Lee et al. [16]	2002	1993–1994	Korea	91/91	76/15 79/12	50 ± 10 65 ± 10	<400 8/60 ≥400 33/31	—	—	—	—	—
Lin et al. [17]	2010	2001–2007	Taiwan	93/73	75/18 53/25	59 ± 15.6 62 ± 12.9	≥400 45/35 <400 48/43	—	8.0 ± 3.3 7.7 ± 4.4	1.8 ± 1.2 1.3 ± 1.3	3.8 ± 0.5 3.3 ± 0.5	S: 49/S: 22 M: 44/M: 56
Luo et al. [18]	2011	2004–2006	China	85/83	70/15 79/4	47.5 ± 12.8 50.9 ± 11.2	238.5 (0–121000) 306.6 (0.6–121000)	60/71 4/1	8.7 ± 3.5 (5–20) 7.8 ± 2.5 (5–15)	15.8 ± 6.9 17.1 ± 7.4	4.05 ± 0.36 4.16 ± 0.40	2 : 35/2 : 32 3 : 20/3 : 11 >4 : 30/>4 : 40
Peng et al. [19]	2012	2002–2007	China	201/402	187/14 374/28	55 (25–75) 55 (23–75)	562.3 598.5	197/389 4/13	≤5 76/178 ≥5 125/224	1.29 1.25	3.68 3.63	1 : 95/1 : 132 >1.106/>1 : 270
Sasaki et al. [20]	1998	1980–1994	Japan	384/534	3.7 4.7	60 ± 8.4 62 ± 7.4	—	—	3.9 ± 3.3 5.4 ± 3.7	0.8 ± 0.4 1.1 ± 0.5	3.7 ± 0.4 3.6 ± 0.5	2-3 : 26/2-3 : 33 <4 : 0/<4 : 2
Sotiropoulos et al. [21]	2009			61/64								
Utsunomiya et al. [22]	2014	2000–2005	Japan	2872/1437	2332/540 1124/313	67 (50, 79) 69 (53, 83)	3491 (15, 16368) 3177 (15, 13605)	2679/1068 193/369	5.8 (1.8, 14) 5.0 (1.4, 13)	0.8 (0.4, 1.5) 1.1 (0.4, 2.3)	4.0 (3.2, 4.7) 3.7 (2.8, 4.5)	1 : 2193/1 : 316 2 : 323/2 : 85 >3 : 126/>3 : 28
Zhong et al. 1 [8]	2014	2000–2007	China	908/351	824/84 326/25	44 (17–78) 53 (19–82)	≥400 434/158 ≤400 474/193	—	8 (4–20)/10 (4–20)	1.3 (0.5–4.0) 1.7 (0.3–22)	3.9 (2.8–4.7) 3.9 (2.2–5.1)	<3845/<3 : 319 >3 : 63/>3 : 32
Zhong et al. 2 [23]	2013	2000–2007	China	257/135	233/24 127/8	46.8 ± 12.0 48.7 ± 12.5	≥400 143/66 ≤400 114/69	—	8.9 ± 3.0 8.8 ± 2.5	14.5 ± 5.3 15.1 ± 8.5	—	S: 199/S: 104 M: 85/M: 31

## References

[1] Mazzaferro V., Chun Y. S., Poon R. T. P. (2008). Liver transplantation for hepatocellular carcinoma. *Annals of Surgical Oncology*.

[2] Mazzaferro V., Llovet J. M., Miceli R. (2009). Predicting survival after liver transplantation in patients with hepatocellular carcinoma beyond the Milan criteria: a retrospective, exploratory analysis. *The Lancet Oncology*.

[3] Sergio A., Cristofori C., Cardin R. (2008). Transcatheter arterial chemoembolization (TACE) in hepatocellular carcinoma (HCC): the role of angiogenesis and invasiveness. *The American Journal of Gastroenterology*.

[4] European Association for the Study of the Liver (2012). EASL-EORTC clinical practice guidelines: management of hepatocellular carcinoma. *Journal of Hepatology*.

[5] Llovet J. M., Schwartz M., Mazzaferro V. (2005). Resection and liver transplantation for hepatocellular carcinoma. *Seminars in Liver Disease*.

[6] Capussotti L., Ferrero A., Viganò L., Polastri R., Tabone M. (2009). Liver resection for HCC with cirrhosis: surgical perspectives out of EASL/AASLD guidelines. *European Journal of Surgical Oncology*.

[7] Torzilli G., Belghiti J., Kokudo N. (2013). A snapshot of the effective indications and results of surgery for hepatocellular carcinoma in tertiary referral centers: is it adherent to the EASL/AASLD recommendations? An observational study of the HCC east-west study group. *Annals of Surgery*.

[8] Zhong J.-H., Ke Y., Gong W.-F. (2014). Hepatic resection associated with good survival for selected patients with intermediate and advanced-stage hepatocellular carcinoma. *Annals of Surgery*.

[9] Chow P. K.-H. (2012). Resection for hepatocellular carcinoma: is it justifiable to restrict this to the American Association for the Study of the Liver/Barcelona Clinic for Liver Cancer criteria?. *Journal of Gastroenterology and Hepatology*.

[10] Huang J., Yan L., Cheng Z. (2010). A randomized trial comparing radiofrequency ablation and surgical resection for HCC conforming to the Milan criteria. *Annals of Surgery*.

[11] Wang J.-H., Wang C.-C., Hung C.-H., Chen C.-L., Lu S.-N. (2012). Survival comparison between surgical resection and radiofrequency ablation for patients in BCLC very early/early stage hepatocellular carcinoma. *Journal of Hepatology*.

[12] Pons F., Varela M., Llovet J. M. (2005). Staging systems in hepatocellular carcinoma. *HPB*.

[13] Choi S. H., Choi G. H., Kim S. U. (2013). Role of surgical resection for multiple hepatocellular carcinomas. *World Journal of Gastroenterology*.

[14] Ho M.-C., Huang G.-T., Tsang Y.-M. (2009). Liver resection improves the survival of patients with multiple hepatocellular carcinomas. *Annals of Surgical Oncology*.

[15] Hsu K.-F., Chu C.-H., Chan D.-C. (2012). Superselective transarterial chemoembolization vs hepatic resection for resectable early-stage hepatocellular carcinoma in patients with Child-Pugh class a liver function. *European Journal of Radiology*.

[16] Lee H.-S., Kim K. M., Yoon J.-H. (2002). Therapeutic efficacy of transcatheter arterial chemoembolization as compared with hepatic resection in hepatocellular carcinoma patients with compensated liver function in a hepatitis B virus-endemic area: a prospective cohort study. *Journal of Clinical Oncology*.

[17] Lin C.-T., Hsu K.-F., Chen T.-W. (2010). Comparing hepatic resection and transarterial chemoembolization for Barcelona Clinic Liver Cancer (BCLC) stage B hepatocellular carcinoma: change for treatment of choice?. *World Journal of Surgery*.

[18] Luo J., Peng Z.-W., Guo R.-P. (2011). Hepatic resection versus transarterial lipiodol chemoembolization as the initial treatment for large, multiple, and resectable hepatocellular carcinomas: a prospective nonrandomized analysis. *Radiology*.

[19] Peng Z.-W., Guo R.-P., Zhang Y.-J., Lin X.-J., Chen M.-S., Lau W. Y. (2012). Hepatic resection versus transcatheter arterial chemoembolization for the treatment of hepatocellular carcinoma with portal vein tumor thrombus. *Cancer*.

[20] Sasaki Y., Imaoka S., Nakano H. (1998). Indications for hepatectomy for hepatocellular carcinoma—what stage of the disease is the best indication for surgery?. *Journal of Hepato-Biliary-Pancreatic Surgery*.

[21] Sotiropoulos G. C., Drühe N., Sgourakis G. (2009). Liver transplantation, liver resection, and transarterial chemoembolization for hepatocellular carcinoma in cirrhosis: which is the best oncological approach?. *Digestive Diseases and Sciences*.

[22] Utsunomiya T., Shimada M., Kudo M. (2014). Nationwide study of 4741 patients with non-B non-C hepatocellular carcinoma with special reference to the therapeutic impact. *Annals of Surgery*.

[23] Zhong J.-H., Xiang B.-D., Gong W.-F. (2013). Comparison of long-term survival of patients with BCLC stage B hepatocellular carcinoma after liver resection or transarterial chemoembolization. *PLoS ONE*.

[24] Williamson P. R., Smith C. T., Hutton J. L., Marson A. G. (2002). Aggregate data meta-analysis with time-to-event outcomes. *Statistics in Medicine*.

[25] Tierney J. F., Stewart L. A., Ghersi D., Burdett S., Sydes M. R. (2007). Practical methods for incorporating summary time-to-event data into meta-analysis. *Trials*.

[26] Parmar M. K., Torri V., Stewart L. (1998). Extracting summary statistics to perform meta-analyses of the published literature for survival endpoints. *Statistics in Medicine*.

[27] Piardi T., Gheza F., Ellero B. (2012). Number and tumor size are not sufficient criteria to select patients for liver transplantation for hepatocellular carcinoma. *Annals of Surgical Oncology*.

[28] D'Amico F., Schwartz M., Vitale A. (2009). Predicting recurrence after liver transplantation in patients with hepatocellular carcinoma exceeding the up-to-seven criteria. *Liver Transplantation*.

[29] Wang Y., Luo Q., Li Y., Deng S., Wei S., Li X. (2014). Radiofrequency ablation versus hepatic resection for small hepatocellular carcinomas: a meta-analysis of randomized and nonrandomized controlled trials. *PLoS ONE*.

[30] Duan C., Liu M., Zhang Z., Ma K., Bie P. (2013). Radiofrequency ablation versus hepatic resection for the treatment of early-stage hepatocellular carcinoma meeting Milan criteria: a systematic review and meta-analysis. *World Journal of Surgical Oncology*.

[31] Ni J.-Y., Xu L.-F., Sun H.-L., Zhou J.-X., Chen Y.-T., Luo J.-H. (2013). Percutaneous ablation therapy versus surgical resection in the treatment for early-stage hepatocellular carcinoma: a meta-analysis of 21,494 patients. *Journal of Cancer Research and Clinical Oncology*.

[32] Ho M.-H., Yu C.-Y., Chung K.-P. (2011). Locoregional therapy-induced tumor necrosis as a predictor of recurrence after liver transplant in patients with hepatocellular carcinoma. *Annals of Surgical Oncology*.

[33] Millonig G., Graziadei I. W., Freund M. C. (2007). Response to preoperative chemoembolization correlates with outcome after liver transplantation in patients with hepatocellular carcinoma. *Liver Transplantation*.

